# AI-Driven Design of High Affinity Biomolecule–Drug Conjugates for Gynecological Cancer Therapy: An Up-to-Date Narrative Review

**DOI:** 10.3390/cancers18111856

**Published:** 2026-06-05

**Authors:** Pankaj Garg, David Horne, Ravi Salgia, Sharad S. Singhal

**Affiliations:** 1 Department of Chemistry, GLA University, NH-19, Mathura-Delhi Road, Mathura 281406, Uttar Pradesh, India; 2 Department of Molecular Medicine, Beckman Research Institute of City of Hope, 1500 E Duarte Road, Duarte, CA 91010, USA; 3 Department of Medical Oncology and Therapeutic Research, Beckman Research Institute of City of Hope, 1500 E Duarte Road, Duarte, CA 91010, USA

**Keywords:** artificial intelligence, antibody–drug conjugates, gynecological cancers, drug resistance, precision oncology

## Abstract

**Background:** Gynecological cancers include collections of cancers with diverse cellular and molecular characteristics that often develop drug resistance, making them treatment-resistant. Biomolecule–drug conjugates (BDCs), especially antibody–drug conjugates (ADCs), have revolutionized the targeted therapy of cancer; however, the creation of these entities has so far been achieved by empirical, resource-intensive design methods. **Objective:** The aim of this review is to critically analyze how AI can be used for the rational design and optimization of high-affinity BDCs for gynecological cancer treatment. **Methods and discussion:** Recent advances in machine learning (ML)- and deep learning (DL)-based methods to predict biomolecule-target binding affinity, structural compatibility, linker stability, payload selection, trafficking in the cell, and biomolecule resistance mechanisms are summarized. The review also explores the possibilities for incorporation of structural, chemical, biological, and multi-omics data to enhance specificity, efficacy, and safety of conjugates. Besides antibody-based systems, AI-assisted design approaches with peptides, aptamers, and hybrid biomolecular systems are also included. This review also highlights parameters and experimental/numerical validation restrictions related to data quality, interpretability of models, regulatory aspects, etc. **Conclusions:** AI-based conjugate engineering is increasingly moving BDC development from a largely ‘trial and error’ approach to a more predictive and data-driven approach. While there are still challenges to be addressed in terms of translations and validations, the potential of AI approaches in the field of precision oncology and the development of more personalized treatment is promising in the context of gynecological cancers.

## Simple Summary

This review summarizes the innovative practice of artificial intelligence (AI) in developing high-affinity biomolecule–drug conjugates to treat gynecological cancer. It discusses how AI-driven approaches overcome limitations of traditional empirical methods by enabling predictive modeling of binding affinity, structural compatibility, linker stability, and payload selection. This article emphasizes integration of multi-omics, structural, chemical, and clinical data to optimize conjugate performance, reduce toxicity, and address tumor heterogeneity and drug resistance. It also explores AI applications in docking, intracellular trafficking, and resistance prediction. Overall, this review presents AI as a powerful tool for advancing precision oncology through rational, data-driven, and personalized therapeutic design.

## 1. Introduction

Gynecological cancers such as ovarian, cervical, endometrial, vulvar, and vaginal malignancies are highly heterogeneous in both biological and clinical aspects, which essentially inhibits the efficacy of a normal therapeutic modality [1]. The diverse genetic changes, epigenetic control, expression profiles on receptors, and composition of the tumor microenvironment (TME) lead to significantly different treatment responses in patients despite having histologically similar tumors. Gynecological malignancies are additionally characterized by distinct biological drivers, including viral oncogenesis in cervical cancer, hormonal dysregulation in endometrial cancer, and extensive genomic instability in ovarian cancer, thereby necessitating disease-specific precision therapeutic strategies. Ovarian cancer is accompanied by a high level of genomic instability and intratumoral heterogeneity, resulting in frequent relapse and low survival rates [2]. Although defined by a more localized presentation at the time of diagnosis, cervical and endometrial cancers undergo dynamic changes in molecular evolution under therapeutic pressure, which propagate the disease and resistance [3].

Precision therapy is about understanding the unique nature of a patient’s tumor and using that understanding to choose treatments that mainly attack cancer cells, while causing lesser harm to healthy tissues. In gynecological oncology, precision is especially important because of the anatomic location of tumors adjacent to important reproductive and endocrine organs, with non-selective toxicity that is associated with significant negative physiological and quality-of-life effects [4]. The role of regulatory therapies is similarly essential in precision treatment of gynecological cancers, and new advances in radiotherapy have contributed to this. Three-dimensional conformal radiotherapy (3D-CRT) makes use of multiple static radiation beams that are shaped depending on the tumor geometry, resulting in a better dose conformity than traditional radiotherapy techniques. It has a limited capacity to adjust doses dynamically; however, it can still over-dose adjacent organs, such as the bladder, rectum, and bowels. On the other hand, volumetric modulated arc therapy (VMAT) provides a continuous radiation delivery using a rotating arc of gantry, at the same time controlling the dose rate, gantry speed and area (MLC) position. This provides improved dose conformation, better sparing of healthy tissue, and shorter treatment time, which makes VMAT a more appealing therapy for the treatment of complex gynecological cancers in which the radiation delivery is more complex [5]. High affinity biomolecule–drug conjugates (BDCs) are therefore an interesting form of precision modality as they combine molecular recognition with targeted cytotoxic delivery. To attain real accuracy, one must not only be precise in target selection but also have a high level of control over binding specificity, internalization kinetics, and drug release dynamics-parameters, which cannot be predicted using the conventional design paradigms.

The traditional formation of BDCs has been grounded on trial-and-error formulations of development, which were carried out through repetitive synthesis, screening, and optimization steps. Although this method has clinically successful results as far as antibody–drug conjugates (ADCs) are concerned, it remains highly inefficient, resource-intensive, and not very scalable. In empirical design, it is difficult to capture the multidimensional nature of the biomolecular interaction with small variations in molecular structure, having significant effects on binding affinity, stability, pharmacokinetics, and toxicity [6]. Empirical methods do not effectively predict the binding behavior, they neglect the conformational flexibility, and offer poor models of intracellular trafficking and linker cleavage. In addition, experimental screening systems usually screen a small portion of potentially achievable conjugation geometries, and large portions of the search space endure to be explored. This restricted discovery serves as a causative element to late-stage failure, unexpected toxicity, and poor therapeutic efficacy. Bevacizumab has proven to be an important anti-angiogenic drug in gynecological oncology in the current arsenal of targeted agents for use. Bevacizumab is a humanized monoclonal antibody that binds to and inhibits the vascular endothelial growth factor (VEGF), which inhibits tumor angiogenesis and reduces tumor vascularization. In combination with chemotherapy, it has shown clinical value in recurrent ovarian, cervical, and endometrial cancers by extending progression-free survival and delaying disease progression. While its therapeutic properties have been recognized, it is still constrained by therapeutic resistance, systemically toxic side-effects, and variable clinical response, with limited availability of predictive biomarkers that would drive a more targeted approach and increased efficacy of the drug [7]. This has necessitated an urgent need for rational and predictive frameworks that can effectively address molecular and biological complexity, characterizing conjugate design.

**Figure 1:**
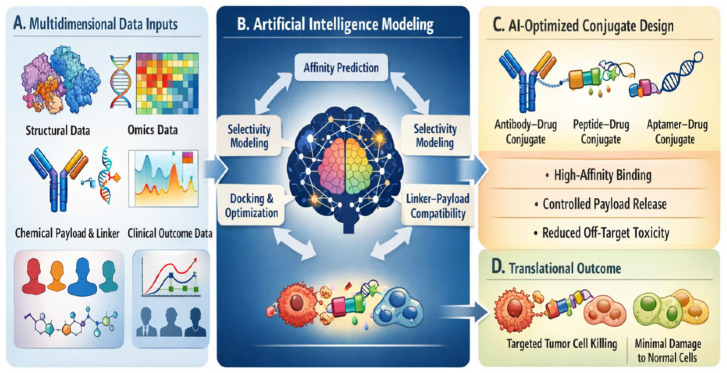
AI-driven workflow for the rational design of high-affinity BDCs in gynecological cancers. The figure exemplifies a comprehensive approach to AI, which integrates structural, multi-omics, chemical, and clinical data to make it possible to systematically design conjugates. The workflow involves identifying the target, predicting its binding affinity, engineering biomolecules, and optimizing linker-payloads. This method, enabled by ML- and DL-based models, empowers the creation of many specific and effective conjugates with better pharmacological properties, decreased non-specific toxicity, and greater adaptability to tumor heterogeneity along with a change in strategy towards elucidating therapeutic design by relying more on prediction than discovery.

The emergence of artificial intelligence (AI) as a revolutionary technology in molecular engineering has provided an effective means to break the constraints of empirical conjugate design. With the combination of machine learning (ML), deep learning (DL), and data-driven modeling, AI-assisted analytical models facilitate the systematic evaluation of high-dimensional data that includes molecular structure, biological interactions, and clinical outcomes [8]. When used in biomolecule conjugates, AI-assisted computational frameworks have demonstrated promising capability in predicting biomolecule–target binding affinity, structural compatibility, linker stability, payload toxicity, and resistance capabilities, which cannot be completed using traditional methods without additional assistance. ML tools with highly developed neural network architectures are able to obtain complex patterns expected to govern biomolecule-target recognition, the characteristics of non-linear correlations, and dynamic changes of conformations [9]. Moreover, AI allows the combination of multi-omics data with structural and chemical data, helping in the development of a personalized conjugate that is designed according to the tumor profile of the individual in gynecological cancers. With continuous growth of computational accuracy, data storage, and analysis, AI will become an inseparable part of precision oncology, redefining the concept of high-affinity BDCs, optimizing them, and translating them into clinical practice [10]. An overview of how AI facilitates the rational design, optimization, and therapeutic performance of BDCs in gynecological cancers is illustrated in Figure 1.

The primary aim of this review is to critically examine the emerging role of AI-assisted frameworks in the rational design and optimization of high-affinity BDCs for gynecological cancers. The secondary aims are focused on discussing recent advances in building affinity matrices, structural docking, linker and payload engineering, intracellular trafficking analysis, resistance prediction, multimodal data integration, and the challenges in translating AI in conjugate development. Beyond that, this review seeks to provide insights into existing challenges, regulatory considerations, and future directions for the use of AI in precision gynecological oncology.

## 2. Data Foundations for AI-Driven Conjugate Design

The successful application of AI to designing high affinity BDCs begins with availability, quality, and acquisition of a diverse range of data modalities. In contrast to traditional drug discovery methods, which tend to utilize distant experimental data, AI-assisted molecular engineering tools need multidimensional datasets that define the structural, biological, chemical, and clinical complexity. Tumor heterogeneity and adaptive resistance are prevalent in gynecological cancer, requiring the need for prevailing databases to advance clinically relevant, generalizable, and predictive AI models for cancer therapy in this field. Table 1 provides a comprehensive overview of the types of biomolecules, design strategies adopted with the help of AI and their translational importance in gynecological cancers.

### 2.1. Structural and Biophysical Interaction Data

AI-based affinity prediction and molecular optimization is based on structural data. The structural restraints of antibodies, peptides, aptamers, and protein scaffolds, singly and as intricate with the molecules of interest (antigens) give vital data on the binding interfaces, conformational freedom, and the energetics of interactions [23,24]. These datasets allow AI models to be trained on the spatial patterns that govern biomolecular recognition, i.e., hydrogen bonding network, electrostatic complementarity, hydrophobic interactions, and steric limits. However, structural data that can be applied to gynecological cancer remains incomplete and biased to the well-studied existing antigens [25]. This weakness is gradually being overcome by AI techniques by using transfer learning, structure prediction, and ensemble modeling, such that models trained on larger datasets of interaction can infer under-characterized gynecological targets. Critically, dynamic structural data describing the conformational change during the binding and internalization process are increasingly regarded as key inputs in next generation AI models [26].

### 2.2. Omics Data for Target Contextualization

The biological context of translating the affinity predictions into therapeutic relevance can be availed by using omics datasets. In gynecologic malignancies, omics-based stratification is especially significant because of disease heterogeneity among the various types of cancer. Chemotherapy resistance and recurrence are caused by high intratumoral heterogeneity, frequent p53 mutations and extensive genomic instability in ovarian cancer. This could include incorporating layers of genomic and transcriptomic differences into AI-based models to better understand clinically relevant and platinum resistance subgroups. However, cervical cancer is mainly linked to human papillomavirus (HPV) infection, and the oncogenes, E6 and E7 are important in cancer promoting and cancer suppressing activities, respectively [27]. The machine-based target identification approaches might help to identify HPV-associated molecular signatures or immune responsive biomarkers. Hormonally controlled, endometrial cancer is one of the most frequently affected pathways that can be altered by the action of AI-driven omics, helping to classify and predict therapies. The cancer-specific omics features reinforce the need for dedicated AI models for the gynecologic context instead of for oncologic data in general.

Genomic and transcriptomic profiles show the patterns of mutation, change of copy number, and expression of antigens in various subtypes of gynecological cancers. Proteomic and glycoproteomic data are used to narrow down the targets of interest by emphasizing post-translational modifications, surface accessibility, and antigen density, all of which directly affect conjugate binding and internalization [28]. The AI-driven combination of omics data allows for the discovery of targets that are context-specific, unscrambling antibodies that are expressed in all tumors and enriched in particular aggressive or drug-resistant tumors. Such contextualization is of great use, especially in ovarian and endometrial cancer where molecular subtypes attain unique therapeutic susceptibility. Using omics-inspired features, AI models can drive beyond the ranks of static affinity prediction towards biologically aware conjugate design [29].

### 2.3. Chemical and Payload-Related Datasets

Chemical data of cytotoxic payloads, linkers, and conjugation chemistries are highly important in the optimization of the therapeutic index of BDCs. These datasets include physicochemical data, stability attributes, metabolism, and toxicity. Training AI models with this type of data can help predict the compatibility of payloads with a particular biomolecular carrier and predict interactions [11]. Also, linker-related datasets facilitate the prediction of cleavage behavior in tumor-relevant conditions, including acidic pH or enzyme rich microenvironment. Chemical intelligence integrated with AI processes is a major step forward in comparison to structure-based practices [30].

### 2.4. Clinical and Translational Outcome Data

Patient response data, progression free survival, rates of toxicity, and rates of resistance offer optimal feedback in the development of AI models. AI models including clinical outcomes can have intricate connections among disease traits and therapeutic reaction and are capable of correctly distinguishing responders and non-respondents [31]. This bidirectional feedback plays a key role in developing AI systems that can provide, as well as support, important outcomes in clinical decision-making. However, challenges such as the inconsistency of quality of clinical data and relative absence of adverse outcomes remain a question. To deal with such concerns, strict validation approaches would have to be employed to reduce bias and enhance the validity of the model [32].

### 2.5. Multimodal Data Integration and Systems-Level Modeling

One of the most impressive capabilities of AI is its ability to take information from multiple types of data in a single predictive model. This system-level approach allows optimization of vital parameters like affinity, specificity, pharmacokinetics, and safety simultaneously and mutually [20]. Such integrative approaches come in handy in the development of conjugates that are both biologically active and clinically feasible, and with high and specific binding capacity. AI-based systems modeling can provide data on the molecular, cellular, and patient levels for gynecological research and treatment. It is a dramatic paradigm change from traditional conjugate engineering to more sensible data and customizable driven approaches [21].

### 2.6. Data Limitations and Standardization Requirement

Although evolving at a rapid pace, AI-based conjugate design is still affected by significant data-related issues. Uncompleted annotation, variability in experiments, and absence of standardized reporting methods inhibit reproducibility and restrict the applicability of the models in general [33]. These problems are especially acute in datasets of gynecological cancer that are most often fragmented and understudied at different institutions. Such constraints will be defeated only after a cooperative endeavor to standardize data generation, annotation, and sharing practices. The development of disease-specific repositories to capture biomolecule-target interactions and conjugate performance data would significantly help in improving AI model training [34].

In spite of the significant achievement gained by AI in BDC engineering, its translational application is limited by data quality, dataset diversity, reproducibly, and clinical validation. While a number of currently accessible structural, proteomic, transcriptomic, and biomolecular interaction datasets remain relatively small and heterogeneous, and heavily skewed toward those antigens with intensive research such as HER2, FRα and EGFR, rarely studied gynecologic tumor subtypes and under-characterized antigens remain comparatively underestimated. This imbalance can lead to an inaccurate prediction and limit the generalizability of the model to patient populations [35]. Furthermore, there are several potential problems associated with reproducibility arising from differences in the sequencing platforms, imaging methods, molecular interaction assays, experiment protocol, and reporting. The lack of complete annotations for the intracellular localization of a protein, resistance-associated pathways, and the data on the response to therapy over time also further reduces the biological reliability of predictions based on AI. In addition, there are several existing AI frameworks that are not fully computationally validated and tested yet to date, and for which there is minimal prospective, prospective clinical, or even gynecological oncology validation [36]. The work presented in this review mainly focuses on the comparatively better developments of the AI-assisted methods based on biomolecule-target affinity prediction, structural interaction modeling, linker optimization, payload optimization, and prediction of resistance-associated therapeutic response. In conclusion, the limitations summarized here point to the importance of having standardized multicenter database sets, harmonized annotation procedures, explainable AI frameworks, and out-focusing the experimental validation approaches needed to ensure reliable and translational use of future AI-assisted conjugate engineering systems.

## 3. Prediction of High-Affinity Biomolecule-Target Binding Using AI Models

**Figure 2:**
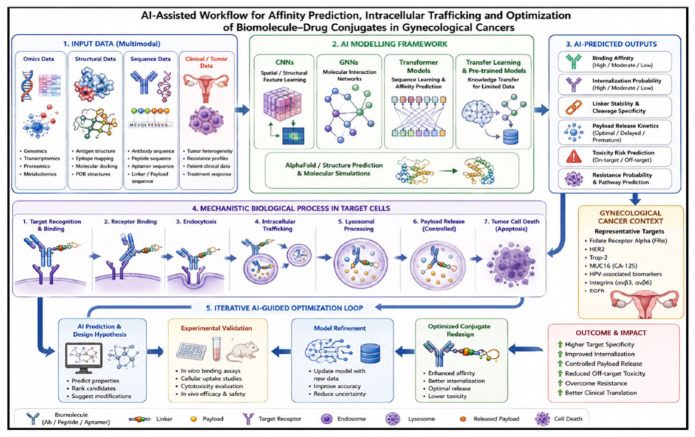
Schematic overview of optimization of BDCs for gynecologic cancer via AI. These multimodal biological data such as omics profiles, structural information, molecular sequence, and tumor-specific clinical data are incorporated into AI systems including the CNNs, GNNs, transformer architectures, and transfer learning systems. These models can be used to predict biomolecule-target affinity, intracellular internalization efficiency, trafficking behaviors, payload release kinetics, toxicity risk and therapeutic response associated with resistance. The workflow also contains mechanisms such as receptor binding, endocytosis, intracellular trafficking, intracellular processing in the lysosome, and the release of a cytotoxic payload, and follows cycles of optimization and experiments guided by AI.

The success of the BDC design depends on the accurate prediction of the binding affinity of a targeting biomolecule with its corresponding tumor-associated antigen [37]. Certain antigens, with heterogeneous expressions that are dynamically regulated, are important in the treatment of gynecological tumors, where just a minor difference in binding strength may result in success or failure of treatment, either by limiting tumor-based interactions or toxicity [38]. Predictive affinity, structural compatibility analysis, linker optimization, and modeling of therapeutic responses are the most remarkable applications of AI in conjugate drug design achieved so far. AI has a special significance in gynecological oncology as the tumor types are highly heterogeneous in their molecular and etiological nature. AI-driven models can be trained using a diverse range of biological signals from various solid and liquid tumor types, such as ovarian cancer, cervical cancer, and endometrial cancer to help identify targets across the context, predict affinities, and optimize therapeutics against cellular targets with resistance mechanisms. These methods might help in advancing advancement of BDCs towards clinically relevant targets like FRα, HER2, Trop-2, etc., and HPV-related biomarkers [9]. An effective solution to this complexity can be provided by AI, which allows predictive modeling of biomolecule interactions that are otherwise difficult to resolve using conventional approaches, as illustrated in Figure 2.

### 3.1. Rule-Based Models for Data-Driven Learning

The primary computational approaches for estimation of biomolecule binding relied on physics-based scoring functions or heuristics or when a rule-of-thumb based on molecular mechanics was used. Although informative, these techniques could not easily address such important factors as conformational flexibility, solvent effects, and context-dependent interactions [39]. On the contrary, AI-based methods, especially ML and DL, denote a radical change to data-driven modeling. Instead of being based on previously known assumptions, these models are trained on the interaction patterns derived from large datasets of known biomolecule-target interactions. These models can identify the nuanced structural characteristics, contributions on the residue level, and the interaction networks that drive binding affinity [17].

### 3.2. Affinity Prediction DL Architectures

DL has been found to be useful, especially with predicting high-affinity biomolecule interactions, because DL can learn complex, hierarchical, and non-linear relationships. Convolutional neural networks (CNNs) are common in the analysis of the structural representations of biomolecule-target complexes in three-dimensional formats to characterize spatial features that determine binding strengths. In the meantime, the transformer-based and recurrent neural network models are efficient in sequence data processing, which means that these models are useful in predicting affinity based on amino acid or nucleotide sequences [14]. These methods allow quick screening of antibody variants, peptide libraries, and aptamer sequences, and allow the discovery of candidates with the best binding properties in gynecological cancer targeting. This flexibility greatly increases the design space to be used in conjugate development [15].

Modern AI-enabled conjugate engineering systems are using a variety of computational architectures as a function of the nature of the biological and structural inputs. To summarize the application areas, convolutions are commonly used for structural imaging and spatial interaction mapping, and a growing number of biological molecular graph representations and predicting the affinity of biomolecules and targets are being made possible by graph neural networks (GNNs) that maintain atomic connectivity and topological relationships [40]. The transformer architecture supports contextual learning dependent on the sequence in protein and peptide databases, and the transfer learning method assists the adaptation of the model used in environments that have few training data for gynecological cancer. Common models can accept inputs such as protein sequences, molecular graphs, biomarker values from omics technologies, docking representatives, and embedding from the AlphaFold code, and can provide outputs such as binding affinity, linker stability, compatibility with the payload, the trafficking behavior of the molecule, and resistance-associated therapeutic response. Structural modeling predictions assisted by AlphaFold have led to a significant boost in accuracy predictions but prediction of reliability of such models for highly dynamic BDC optimization and prediction of intracellular interaction remains a subject of ongoing investigation. Typically, sets of structural data and interaction data that are experimentally annotated are used for model training, and models are frequently evaluated for their prediction accuracy, loss minimization, the area under curve (AUC), and generalizability to independent validation sets [41]. The potential challenges like limited benchmark datasets, uncertainty quantification, and varying structural prediction accuracy have remained, limiting wider translation of AI-driven conjugate optimization frameworks (Table 2).

### 3.3. Binding Kinetics and Affinity Landscape Modeling

**Figure 3:**
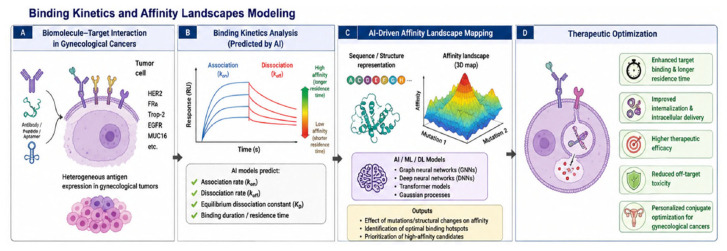
AI-driven binding kinetics and affinity landscape modeling for BDC optimization in gynecological cancers. Schematic representation of AI-assisted binding kinetics and affinity landscape modeling in BDC engineering for gynecological cancers. AI-based frameworks that combine biomolecular sequence, structure and interaction data to predict association/dissociation kinetics, optimize binding affinity, and predict therapeutic performance. In addition, AI-driven affinity landscape mapping allows for logical optimization of biomolecule–target interactions to achieve more targeted delivery into cells and more specific therapeutic design.

Binding affinity can normally be described by a single equilibrium constant, but kinetic properties of association and dissociation rates are closely related to therapeutic efficacy. Contemporary AI models are becoming increasingly capable of considering these dynamic features, and these models are able to predict not only binding strength, but also duration of interaction as illustrated in Figure 3.

Such observations are most widely applied using dynamic ideas in gynecological tumors, where the distribution of antigens may be heterogeneous, and the transient interactions may still render the successful delivery of a drug [42]. The affinity landscape modeling that is driven by AI also improves design strategies through mapping the effects of sequence or structural changes on the binding behavior in a continuum of possibilities. This way, AI is transformed into a predictive instrument rather than an informative and rational platform to biomolecule design [43].

### 3.4. Predicting Selectivity and Off-Target Interactions

Selectivity must not be lost, even when high-affinity binding is achieved. System toxicity can be the result of non-selective reactions, which can challenge the clinical potential of BDCs. The solution to this problem proposed by AI models is to concurrently consider the binding potential of many targets in order to predict selectivity profiles at an earlier stage of the design process [18]. AI-assisted selectivity prediction holds a special place in gynecological cancer applications, where most of the tumor-associated antigens are structurally related to normal tissue proteins. By acquiring minor differences in binding interfaces, AI models assist in prioritizing biomolecules that bind in favor of malignant cells, thus enhancing safety factors and therapeutic accuracy [44].

### 3.5. Transfer Learning and Learning with Limited Data

One of the enduring problems in gynecological oncology is that the available data on interaction issues with rare or newly identified targets is often poor. Transfer learning has emerged as a serious tool in solving this limitation [45]. Training using only benchmark images is insufficient to accurately determine the stage of the disease. In ovarian and cervical cancers, previous cancer studies have shown that AI-based molecular profiling is useful for identifying the molecular subtype-specific treatment targets and resistance-related biomarkers [44,45]. This is particularly desirable when dealing with novel biomarkers and patient targets in gynecological cancer. Transfer learning enables AI models to take advantage of the generalization of knowledge on biomolecule recognition and adapt to the specifics of the molecular setting of tumors. Consequently, predictive-based affinity is readily available in a broader number of clinical contexts based on AI [46].

### 3.6. Human-Centered Interpretation of AI Predictions

**Figure 4:**
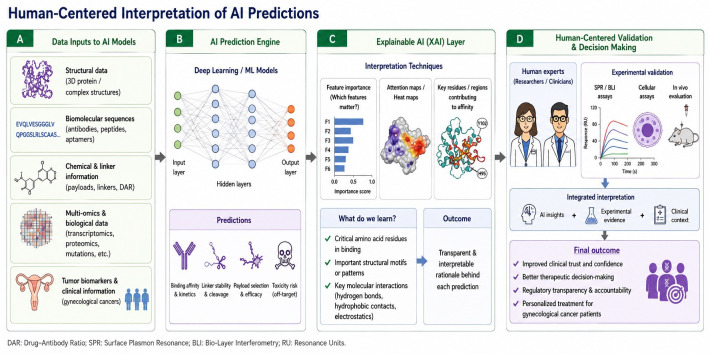
Human-centered interpretation and explainable AI frameworks in BDC prediction models. Illustration of human-centered and explainable artificial intelligence (XAI) approaches in BDC development. AI models integrate structural, biological, and molecular datasets to predict therapeutic performance parameters such as binding affinity, linker stability, and toxicity. Explainable AI frameworks identify the critical molecular features driving model predictions, thereby improving interpretability, experimental validation, clinical trust, and translational applicability in gynecological oncology.

The development of AI models has great predictive capabilities, but interpretation and trust are prerequisites for their clinical implementation [47]. Attempts to render the mechanism of AI prediction have led to the emergence of explainable AI systems (Figure 4), which expose significant residues, structural patterns, or molecular features that emerge as the drivers of affinity predictions. This interpretability is necessary to fill the gap between computational output and experimental validation [48].

### 3.7. Implications for Conjugate Design in Gynecological Cancers

The use of AI-based affinity prediction models will have far-reaching consequences on the design of BDCs in gynecological cancers. AI enhances high-quality outcomes in terms of early detection of targeting ligands, high affinity, and high selectivity, thus lowering the experimental workload and shortening development times. Moreover, it is possible to optimize conjugates rationally using AI-based reasoning in various tumor environments, such as resistant and recurrent disease [49]. The change is a major step towards coming up with more specific conjugates, which can combat the biological sophistication of gynecological malignancies. These are the stage-by-stage contributions of AI helping to enhance design efficiency and therapeutic outcomes, which are comprehensively summarized in Table 3.

## 4. AI-Assisted Structural Docking and Conjugate-Oriented Molecular Optimization

The ultimate success of a BDC is also determined by the structural compatibility between the targeting biomolecule and the corresponding tumor-associated antigen. The optimal structural alignment is especially hard to achieve in gynecological tumors, with antigen conformation, accessibility on the surface, and microenvironment differing greatly across sites [59]. As a dynamic and adaptive learning process, molecular docking has evolved in the context of AI, moving beyond its earlier static, approximation-based basis to depict the complexity of biological systems with greater accuracy. The combination of structural prediction and conformational sampling with the aid of AI, known as AI-assisted docking, encompasses rational and conjugate-aware molecular optimization within unified workflows [60].

Conventional docking methods usually fail with large and complicated biomolecules like antibodies and peptides, particularly in cases where the structure of the antigens is incomplete or regulated. Docking frameworks made using DL are capable of predicting binding poses and take into consideration induced fit, solvent effects, as well as side-chain rearrangements, which are critical to the modeling of biomolecule-target interactions in gynecological cancers. The ability allows the replacement of simplistic lock and key assumptions with more realistic models of binding behavior [61].

It is interesting to note that AI-assisted docking is increasingly becoming more and more conjugation-specific in its constraints, which tend to be ignored in traditional structural modeling. Linker and cytotoxic payload conjugation may have a considerable effect on biomolecular conformation, steric accessibility, and binding orientation. The effects can be captured by AI models trained over conjugated systems, which forecast the influence of the length of linker, where the target is bound or where it is unbound, and the size of the payload in target engagements [62]. The other significant benefit of AI-based structural optimization is that it effectively examines large design spaces. Instead of considering each variant one at a time, AI models can generate and rank thousands of structural candidates, finding those that maximize binding stability in addition to desirable pharmacokinetics. This method considerably hastens optimization of antibodies, peptides, and aptamers and minimizes the use of the resource-consuming experimental screens [16]. More advanced AI models now involve learning with molecular dynamics and the idea of modeling the dynamic behavior of biomolecule target complexes, besides the previous static structure prediction. These models acknowledge that the binding phenomenon is not absolute but varies on the conformational flexibility and interactions between the membrane and environmental factors like pH and ionic strength [19]. These variables enable AI to locate binding modes to be consistent within the physiological and tumor-based setting, and, consequently, enhance the translational relevance. AI-aided docking and optimization for structure is an AI-based paradigm shift in BDC engineering. With proper support of some auxiliary tools of structural realism, conjugation awareness, and high throughput exploration, a rationally designed AI can be used with the help of to build structurally robust, biologically active, and clinically viable conjugates. Gynecological cancers treated with the use of this technique promote precision-guided targeted therapies and can eliminate tumor heterogeneity and resistance to treatment [63].

## 5. AI-Guided Linker and Payload Engineering for High-Precision Conjugates

**Figure 5:**
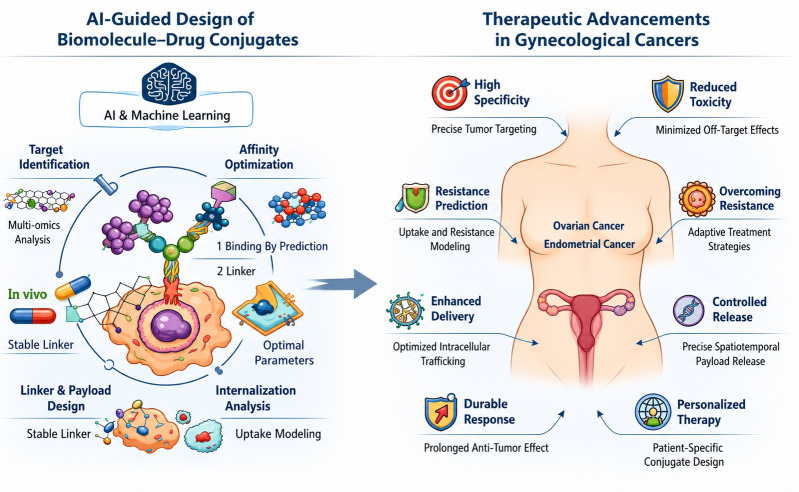
Design and clinical translation of BDCs with the direction of AI to treat gynecological cancers. The figure shows a combined AI-based system that involves identifying the target using multi-omics analysis, optimization of affinity, design of linker-payload, and internalization analysis to allow rational conjugate engineering. Optimization of binding, stability, and intracellular delivery can be achieved with the help of ML models. This design pipeline can be translated into better therapeutic outcomes, such as increased tumor specificity, less non-target toxicity, predicting and mitigating resistance, improved cellular uptake, long-term anti-tumor effects, and personalized and patient-specific treatment plans in gynecological tumors.

The therapeutic efficacy of drug–biomolecule conjugates is not just about ensuring high affinity to the target, but are about being smartly designed, where the type of bio-conjugation determines its stability, release and the release’s effect on its cytotoxic capacity [12]. In the realm of considering linker design and payload selection, AI-driven approaches have been helpful when it comes to predictive modeling and optimizing for data-driven linker chemistry and payload compatibility, rather than a trial-and-error approach. This hybrid approach aims to utilize AI to improve the efficiency of linker chemistry, payload selection, cell- and intracellular processing, and ultimately therapeutic specificity and safety as shown in Figure 5.

### 5.1. AI-Driven Linker Design and Controlled Payload Release

The molecular linker between targeting biomolecule and cytotoxic payload has to be designed in such a way that it controls systemic stability and is useful in the delivery of the payload specifically toward tumors. ML models can predict the behavior of various designs of linkers based on a database of linker chemistry and the enzymatic cleavage data. The in vivo stability can predict how the linker will be used in vivo in physiological and tumor-relevant environments [50]. AI-guided linker selection enables context-dependent conjugate engineering in gynecological cancers, which might exhibit a modified pH, redox potential, and protease activity. With the combination of chemistry structure representation and absolute reporting of the biological reaction, AI systems can predict the kinetic performance of linker cleavages and suitable sites in which drugs are least likely to be released. Furthermore, AI-informed methods contribute to the generation of stimuli-responsive linkers depending on the distinctive biochemical topographies of gynecological tumors [64]. Despite these great advances, existing AI tools for linker optimization are still limited in scope and widely diverse in the range of experiments they can be applied to, and, relatively speaking, fewer comprehensive experimental validations have been performed in gynecological cancer systems. For this reason, the relational validity and extent to which the computation methods are applied to other studies to predict linkers remains to be validated in experiments.

### 5.2. Payload Selection, Compatibility, and Safety Optimization

Selecting the cytotoxic payload is essentially key to the BDC potency and safety profile. The analysis of payload-related data with the use of AI would allow for systematic analysis of drug potency, mechanism of action, physiochemistry, and toxicity risks. Instead of employing cytotoxic strength as the main criterion to choose payloads, AI models consider its compatibility with the targeting biomolecule, linker chemistry, and its expected intracellular trafficking pathways [6]. As an example, it is possible to use AI models that determine whether the mechanism of action of a payload can match the proliferative status or repairing capacity of certain gynecological cancer subtypes. These context-dependent optimization strategies can enhance therapeutic success and minimize therapeutic failure due to resistance. However, it is difficult to predict what the payload will do inside cells as there can be poorly defined biological data and different pharmacodynamics responses in tumors [52].

### 5.3. Integrated Linker-Payload Co-Optimization Using AI

Among the largest innovations that are facilitated by the process of AI is the ability to optimize both linkers and payloads as a single system and not as separate entities. Multitask AI models are capable of comparing the effect of changes in the chemistry of linkers in terms of payload stability, intracellular release, and cytotoxicity. This holistic approach is an essential component of preventing trade-offs in design that would lead to the loss of conjugate performance [65]. Practically, using AI-conjugation to co-optimize conjugates provides a designer with the ability to design conjugates that fulfill a concrete clinical goal, e.g., the desire to increase tumor penetration in large ovarian tumors or to decrease the levels of non-specific toxicity in maintenance treatment. However, existing co-optimization models are largely computational, or preclinical and early-stage models, with very few prospective biological validations in gynecological oncology research. Moreover, the complexity of tumor heterogeneity, variability of intracellular transport, and changes in the microenvironment of the cells keep the predictive modeling of conjugate behavior at a big challenge in scaled-up predictive approaches [66].

## 6. AI-Enabled Prediction of Cellular Internalization, Intracellular Trafficking, and Resistance Dynamics

The high affinity of a BDC is crucial because the target binding not only dictates the ultimate activity but also the capacity to be internalized by the cell more effectively, the correct intracellular trafficking, and the release payload of malignant cells. These latter downstream biological processes are highly heterogeneous and are likely to be the areas most critical to therapeutic resistance in gynecological cancers. AI is becoming a staple in unraveling intricate cellular processes to deliver predictive knowledge that can carry conjugate design far beyond the much longer surface-reactive binding affinities to more real functional action in biology. Despite a considerable progress in the development of tools for predicting cellular transport and behavior, the algorithms of intracellular trafficking behavior, endosomal escape, kinetics of payload release and resistance evolution remain highly challenging to accurately predict due to the dynamic nature of the internal microenvironment and the complexity of the biological interactions involved that is partly unresolved in the experiment. As such, the majority of contemporary AI-driven intracellular transport and resistance prediction models are still in the exploratory or early preclinical stages [67].

### 6.1. Predicting Internalization Pathways and Endocytic Efficiency

After recognition of the antigen, BDCs must undergo endocytosis so that they can release their cytotoxic drugs into the cell. The effectiveness and route of internalization, i.e., clathrin-mediated endocytosis, caveolae-dependent uptake, or microphage pinocytosis, is determined by antigen properties, biomolecule structure, and cellular environment. AI models, trained on imaging, receptor science, and inner cell studies of dynamic interactions can forecast the behavior of various conjugate design–cellular uptake interactions [53]. These types of predictive data may be beneficial to decide on suitable biomolecular format and target antigens for gynecologic cancer where the expression level and dynamics of receptors vary considerably amongst tumor types and disease states. Multi-dimensional cell data, possibly analyzed by AI, can further optimize their uptake and delivery efficiency into the cell, and could still be used as a complement [68].

### 6.2. Modeling Intracellular Trafficking and Payload Release

Conjugates are then transported through complex intracellular pathways after the endocytosis transition and release the payload to the right target cells and tissues. The numerous parameters (enzymatic activity, type of linker chemistry, properties of the payload, etc.) take different weights; and are important in different contexts such as avoiding endosomal escape, lysosomal degradation, and cytosolic delivery. These can be mimicked using AI constructs that combine both cellular and biochemical molecular characteristics to more accurately forecast cellular fate [54]. AI-based modeling has a tremendous strategic advantage when it comes to gynecological cancers, whereby therapeutic resistance is often associated with disrupted intracellular trafficking pathways. These AI-based methods allow optimizing linker responsiveness and payload stability through forecasting the most likely sites and times of drug release. This predictive accuracy is more likely to ensure that cytotoxic agents arrive at their desired intracellular destinations and thus improve therapeutic efficacy with a reduction in unwanted adverse effects [69]. In spite of significant progress that has been made in understanding intracellular trafficking using AI tools, the precise modeling of endocytic routing, escape of the lysosome, kinetics of payload delivery to endosomes, and the transport and metastases between different intracellular compartments is still a very challenging task due to the scarcity of standardized long-term trafficking datasets, and the large inter- and intra-species variability of tumor cells. Robust models of intracellular transport processes are strongly affected by the local dynamic environment, cell heterogeneities, and time-varying changes in cellular signaling that are not fully accounted for by existing models in this field [70].

### 6.3. Anticipating Resistance Mechanisms and Adaptive Tumor Responses

Even the latest targeted therapy has failed to address one of the leading challenges, namely resistance in treating gynecological malignancies. Antigen downregulation altered endocytic pathways, enhanced drug efflux, or amplified DNA repair can all enable tumors to evade conjugate-based treatments. The patterns of adaptive tumor behavior can be found in AI models that are trained on longitudinal data, which incorporates treatment response and resistance emergence [71]. Anticipating resistance strategies has allowed AI to be proactive in conjugate design by allowing alternative targets to be selected, dual-targeting biomolecules to be used, or payloads that circumvent the well-recognized mechanisms of resistance to be used. Accurate prediction of therapeutic resistance and tumor evolution is limited by the small number of longitudinal molecular observations and the dynamic nature of tumor ecosystems. The mechanisms of resistance to therapy can be complex for gynecological cancers, with antigen modulation, rewiring of intracellular signaling, epigenetic resistance, and microenvironmental-associated therapeutic escape being some of the mechanisms that are poorly understood, and even less characterized, at the computational level. Thus, the real-world predictive power of novel AI models for resistance-associated patterns, as presented here, when confronted with clinically changing tumor conditions is a space for future investigation [55].

## 7. Translational Challenges, Clinical Integration, and Regulatory Considerations for AI-Designed Conjugates

Though AI has shown strong potential in the design of high-affinity biomolecules in drug conjugates, there still exist a broad range of challenges in translating computational innovations into clinically approved therapies. These issues must be addressed to extend AI-assisted conjugate engineering beyond theoretical capability toward meaningful therapeutic applications. The vast majority of existing ML models have been trained on comparatively small and non-representative datasets, or on datasets that do not represent gynecological tumors as a whole. Furthermore, many existing AI frameworks are not yet extensively and comprehensively validated in clinical practice or experiments. An additional challenge is model interpretability, which poses a significant question for systems that use DL as a “black box” approach that do not have a high degree of biological transparency. In addition, differences in molecular data types, experimental approaches, expression levels of the biomarker genes, and assay procedures hinder reproducibility and external validation between different studies [57].

Although computational biomolecule engineering has emerged as a promising new area in which AI tools can be applied, there are few examples of AI-assisted BDCs that have been extensively validated in terms of biology or clinically tested in the context of gynecological oncology. Presently, most available studies are still based on preclinical affinity prediction, virtual screening, optimization of the structure and resistance-associated therapeutic modeling rather than clinically proven therapeutic deployment. While AI-driven tools have shown promising potential in target selection, linker optimization, and molecular interaction predictions, few treatments in the field of gynecological cancers have been directly compared with an engineer approach. Furthermore, due to tumor heterogeneity, variations in trafficking behavior, changes in the microenvironment, or incompleteness of the datasets, there are potential discrepancies between affinity computed through in silico methods and biological activity seen in experiments [72]. The mutually reinforcing constraints all underscore the ongoing clinical need for strict experimental testing, benchmarking standards, and clinically relevant AI-aided therapeutic innovation pathways.

AI-based design methods have several potential advantages over traditional empirical conjugate generation methods: quicker virtual screening, faster discovery of biomolecule–target interactions, optimization of linker and payload for maximum interaction, minimizing the need for repetitive experimental synthesis, and screening cycles. Some recent research indicates that models supported by ML have the potential to achieve better accuracy on binding affinities, multidimensional and structural compatibility, as well as resistance-associated therapeutic response. Many of the tools or methods available today that leverage AI are still exploratory or preliminary preclinical studies; however, there are still evolving standardized metrics measuring quality of prediction, time, and clinical efficacy. For this reason, AI-driven frameworks can be used in tandem with and complement traditional conjugate engineering approaches, but they have not been definitively proven to work better than empirical strategies [65]. A comparative and critically balanced perspective of conventional therapies, traditional conjugates, and emerging AI-assisted BDCs is presented in Table 4.

### 7.1. Bridging Computational Predictions with Experimental and Clinical Validation

The biggest obstacle is the inability to bridge the gap between AI-based predictions and the testing of the hypothesis. Although AI models put forward the most efficient conjugate designs in very short time, these predictions should be considered after thorough in vitro and in vivo research. Uncertainties in computational predictions and biological reality may come about because of incomplete data, biological variation, or a physiological factor that was not modeled [73]. Such discrepancies are of special importance in gynecological oncology, where TME and individual aspects of the patients are of particular relevance. In response to this, coupled AI designing and repetitive experimental feedback-integrated workflows are becoming more widely used. Through collaboration between computational scientists, chemists, and clinicians, such workflows ensure that AI-based innovations are based on biology and clinical reality [74].

### 7.2. Clinical Implementation and Patient Stratification

The clinical use of AI-designed BDCs needs to be carefully considered in terms of patient selection and treatment stratification. A particular conjugate is not necessarily effective for all patients with gynecological cancers, even when they present with the same antigen [75]. Logistically and ethically, such AI-helped stratification into clinical practice is difficult. Data availability, data standardization, and data interpretability are key factors to being trusted and adopted by clinicians. It is likely that explicit AI models demonstrating effective justification of patient-based decisions will be accepted in healthcare practice. In addition, regulatory standardization for AI-assisted therapeutic design remains insufficiently established, particularly regarding dataset transparency, model reproducibility, validation standards, and clinical interpretability requirements [58].

### 7.3. Regulatory and Ethical Considerations

The novel concept of regulatory clearance of an AI-driven therapeutic carries a number of complexities beyond those of standard drug development approaches. The regulatory authorities are looking for the outline of the design justification, the safety profile and manufacturing uniformity. There are several important contributions of AI systems in the decision-making process associated with molecular design, along with model validation, model validation reproducibility, and the system’s accountability as the system grows [76]. It is also crucial to modify the regulatory landscape to ensure that the innovation with AI does not impair high standards of evaluation in gynecologic cancer care, with patient safety as the measure of their bottom line. Ethical issues such as the privacy of information and the bias of the algorithms should be proactively taken care of, and the capacity of AI-assisted therapies should be equally accessible to all [77]. Although AI-powered molecular modeling apps have shown great advancement, the majority of existing BDC optimization frameworks are still in their computational and preclinical stages with not a single one validated in prospective clinical scenarios relevant in gynecological oncology.

## 8. Future Perspectives: AI as a Catalyst for Precision Conjugate Oncology in Gynecological Cancers

AI is poised to reshape the landscape of BDC development not as a secondary calculating tool, but as an innovator of accurate oncology. In gynecological malignancies, where the issues of biological complexity, resistance to therapies, and inter-patient variability have been persistent, AI has been linked with the opportunity to offer a framework that would enable the alignment of clinical decision-making process and molecular intuition [78].

### 8.1. Toward Personalized and Adaptive Conjugate Therapies

The personalization of BDCs based on the profiles of patients is an interesting direction that can be developed in the future. The choice of the best targets, biomolecule carriers, and payloads to use with each patient can be directed by AI systems that incorporate genomic, proteomic, and clinical information. Personalization has the potential to reduce toxicity in gynecological oncology, where progression of disease and therapeutic response are widely divergent [56]. Through the constant adaptation of recommended changes to the targeting strategies or choice of payload based on the results of treatment and the patterns of resistance, the AI models can assist in recommending changes throughout the disease management process [13].

Internet of Things (IoT) is an emerging technology that has revolutionized oncology by connecting patients, healthcare systems, and medical devices in real-time. Wearable sensors and smart monitoring systems facilitated by IoT technology can monitor important physiological information, treatment-related side effects, activity level, and adherence to medication through the treatment of chemotherapy or radiotherapy in gynecological cancer. This data collection at the right time may assist in detecting those types of issues early, make better individualized treatment modifications, and provide greater supportive treatment [79]. Furthermore, an Internet of Things-driven system connected to AI analytics and EHR has the potential to enhance the remote care of patients, such as strict follow-up for women undergoing treatment for ovarian, cervical, and endometrial cancers, and care for a longer period of time. To conclude, the use of IoT has promise to improve patient engagement, timely interventions, and precision gynecological oncology medical care [80].

### 8.2. Expanding Design Paradigms Through Multi-Targeting and Hybrid Systems

The future of AI-based conjugate design will possibly shift to multi-targeting and hybrid systems that will confront the challenge of tumor heterogeneity in a more effective manner, rather than employing a single-target design. AI can discover antigen combinations that are complementary and develop biomolecule structures that can interact with multiple targets in parallel or in sequential order [22]. These approaches are especially applicable in gynecological malignancies, during which heterogeneous expression of the antigens tends to compromise single-target therapies. Optimization of these complex systems through AI can result in a balance between binding affinity, selectivity, and pharmacokinetics to make the rational design of new generation conjugates possible [81].

### 8.3. Building a Collaborative and Responsible AI Ecosystem

The realization of AI success in conjugate oncology lies in the future, not just in the further technological development, but also in the introduction of collaborative and ethical systems. AI-driven designs will rely on cross-disciplinary cooperation between computational scientists, biologists, chemists, clinicians, and regulatory professionals who will be needed to convert AI-driven designs into clinical reality [36]. The open data disclosure, the standardized validation metrics, and openly reported activities will further enhance the quality of AI-directed research. The most significant aspect of the practice is to trust AI systems with treating cancers in gynecology because treatment plans have severe personal and social impacts [82].

Although several proposed systems based on AI have demonstrated promise in optimization of BDCs, practical aspects involving what has been achieved and what can be achieved with translation, and ongoing conceptual therapeutic applications must be taken into account. Most of the clinically proven conjugates are developed by traditional biomolecular engineering approaches that were validated in the lab, while AI-driven approaches are primarily employed for optimization, prediction of affinity, virtual compound screening, and in molecular modeling. More advanced strategies like real-time adaptive conjugate redesign, or patient-specific resistance-guided therapeutic adjustment, and dynamic evolution of AI-driven treatment optimization, however, are largely investigational methods, and are not yet accepted in the day-to-day treatment of oncology patients. Prospective validation, FDA drug approval, biological interpretability in the context of existing/available knowledge and computational standardization, and clinical integration are significant implications or concerns for the adaptable AI therapeutic systems that are not yet clinically feasible [83].

The importance of regulatory and ethical considerations for the responsible implementation of AI-aided conjugate design cannot be overstated in this context. The factors that are important include explainability and transparency of the models, reproducibility across datasets and institutions, preclinical and clinical validation standards, intellectual property of AI-generated molecular entities, and accountabilities for the use of AI in therapeutic decisions. Furthermore, regulatory approaches are undergoing adaptation, such as from the U.S. FDA and the European Medicines Agency (EMA), increasingly oriented towards risk-based assessment, clinical validation, and transparency on reporting the results of AI-based biomedical technologies. The applications of AI-assisted BDC engineering in gynecological oncology will only be truly realized by working through a variety of governance requirements that goes beyond (but encompasses) the computational innovation [84].

## 9. Conclusions

The game changer is AI, which facilitates the development of highly effective BDCs for the treatment of gynecological cancer. The interface between clinical need and molecular complexity makes therapeutic approaches to traumatic situations precise, optimally rational and adaptive, with the aid of AI. The next step in the establishment of a new era of accurate oncology might not result from empirical evidence but rather informed and patient-guided design, fueled by AI-based conjugate engineering as data ecosystems become more robust and collaborative models gain greater power. AI in BDC engineering offers significant promise in the realm of gynecological oncology for such applications as drug affinity prediction, linker optimization, and designing resistant, superior therapeutics, yet many of the cutting-edge immunoconjugate engineering strategies and applications described in this review are still at the preclinical or conceptual level. As such, further extensive experimental testing, regulatory evaluation, and future clinical trials are needed before clinical routine, and broad translation is implemented in the field of gynecological cancer therapy.

## 10. Clinical Impact of AI-Driven High-Affinity BDCs in Gynecological Cancer

• Enhances precision oncology by enabling patient-specific design of BDCs, improving therapeutic efficacy while minimizing non-specific toxicity and preserving healthy reproductive tissues.
• Accelerates clinical translation by reducing trial-and-error development, enabling faster identification of optimal candidates with better safety, stability, and efficacy profiles in gynecological cancers.
• Improves treatment outcomes by predicting resistance mechanisms and optimizing intracellular delivery, leading to more durable responses and effective management of recurrent and heterogeneous tumors.

## Figures and Tables

**Table 1:** AI-guided BDC design strategies in gynecological cancers.

Biomolecule Type	Typical Targets in Gynecological Cancers	AI Models Used	Representative AI Platforms/Datasets	Key Design Parameters Optimized	Functional Advantages	Representative Applications	Translational Status	References
Monoclonal antibodies (ADCs)	HER2, FRα, Trop-2, MUC16, EGFR	Deep neural networks, GNNs, AlphaFold-based structural prediction	AlphaFold, Protein Data Bank (PDB), BindingDB, DeepChem	Antigen–antibody affinity, epitope mapping, linker stability, drug–antibody ratio (DAR)	High specificity, prolonged circulation, strong internalization	Ovarian & endometrial cancer ADCs (e.g., FRα-targeted systems)	Several FDA-approved; next-generation AI-optimized ADCs in preclinical stage	[5,6,11,12,13]
Antibody fragments (Fab, scFv, nanobodies)	HER2-low tumors, stromal antigens	AI-guided protein engineering, sequence–structure modeling	Rosetta AI, AlphaFold-Multimer, SAbDab database	Binding kinetics, size reduction, penetration efficiency	Improved tumor penetration, reduced immunogenicity	AI-designed nanobody–drug conjugates	Preclinical	[14,15,16]
Peptide–drug conjugates (PDCs)	Integrins, LHRH receptor, folate receptor	ML, peptide–target docking algorithms	Peptide Atlas, Auto Dock, Deep Purpose	Peptide affinity, stability, enzymatic cleavage site selection	Small size, rapid internalization, low immunogenicity	Ovarian cancer-targeted PDCs	Preclinical–early clinical	[14,17,18]
Aptamer–drug conjugates (ApDCs)	EpCAM, nucleolin, VEGF	AI-based SELEX optimization, molecular dynamics simulations	SELEX datasets, RNA Composer, molecular dynamics simulation platforms	Aptamer folding stability, binding energy, serum stability	High specificity, easy synthesis, tunable chemistry	Cervical and ovarian cancer ApDCs	Preclinical	[15,19]
Protein scaffold-based conjugates	TME markers	AI-guided scaffold redesign	Rosetta, Protein MPNN, FoldX	Structural rigidity, binding orientation	Modular design, enhanced stability	Experimental systems	Exploratory	[16,19]
Multi-biomolecule conjugates	Dual antigens (HER2 + FRα)	Multi-task learning AI models	Multi-omics integration platforms, TCGA, ensemble AI frameworks	Dual-affinity balancing, payload synergy	Overcomes heterogeneity, reduces resistance	Dual-target gynecologic conjugates	Emerging	[20,21,22]

ADCs, antibody–drug conjugates; AI, artificial intelligence; ApDCs, aptamer–drug conjugates; DAR, drug–antibody ratio; EGFR, epidermal growth factor receptor; EpCAM, epithelial cell adhesion molecule; Fab, fragment antigen-binding; FDA, Food and Drug Administration; FRα, folate receptor alpha; GNNs, graph neural networks; HER2, human epidermal growth factor receptor 2; LHRH, luteinizing hormone-releasing hormone; MUC16, Mucin 16; PDCs, peptide–drug conjugates; scFv, single-chain variable fragment; SELEX, systematic evolution of ligands by exponential enrichment; VEGF, vascular endothelial growth factor.

**Table 2:** Representative AI methodologies in BDC design for gynecological cancers.

AI Methodology	Typical Input Data	Predicted Output	Application in Conjugate Design	Representative Use Case
Convolutional Neural Networks (CNNs)	3D protein–ligand structural grids, docking poses	Binding affinity, spatial interaction patterns	Structural feature extraction for antigen–biomolecule binding prediction	ADC binding site optimization in ovarian cancer targets
Graph Neural Networks (GNNs)	Molecular graphs (atoms, bonds, interaction networks)	Affinity scores, molecular interaction fingerprints	Modeling biomolecule–target interaction topology and binding energy	FRα/HER2 targeting antibody optimization
Transformer-based models	Protein sequences, peptide libraries, SMILES strings	Sequence-based affinity prediction, binding probability	Context-aware sequence learning for biomolecule engineering	Cervical cancer HPV-related antigen targeting
Transfer Learning models	Pretrained protein–ligand datasets + limited cancer-specific data	Adapted predictive models for new targets	Enables model training in low-data gynecological cancers	Rare ovarian tumor antigen prediction
AlphaFold-assisted structural modeling	Amino acid sequences	3D protein structures	Structural embedding for docking and affinity prediction	Endometrial cancer receptor modeling
Ensemble Learning models	Multi-omics + structural + chemical data	Integrated prediction of efficacy/toxicity	Multi-parameter conjugate optimization	Multi-target gynecological conjugates

**Table 3:** Role of AI across the entire BDC development pipeline.

Conjugate Development Stage	Conventional Approach	AI-Based Strategy	Key Algorithms/Models	Representative Tools/Datasets	Outcomes Improved	Impact on Gynecological Cancer Therapy	References
Target identification	Manual biomarker screening	Multi-omics AI integration	Random forest, DL, network biology	TCGA, GEO, cBioPortal	Accurate tumor-specific target discovery	Identification of novel ovarian and endometrial cancer antigens	[2,20,29]
Affinity prediction	In vitro binding assays	AI-predicted binding energy and kinetics	GNNs, molecular docking AI	AlphaFold, PDBbind, DeepChem	High-affinity selection before synthesis	Reduces experimental failure rate	[14,24,37]
Biomolecule engineering	Trial-and-error mutagenesis	AI-guided sequence optimization	Protein language models, transformer networks	ESM protein language models, Rosetta	Improved stability and specificity	Generation of next-generation antibodies and peptides	[14,15,16]
Linker design	Empirical chemistry-based approaches	AI-predicted cleavage specificity	Molecular dynamics + ML	Auto Dock, molecular dynamics simulation datasets	Controlled intracellular release	Reduced non-specific target toxicity	[12,50]
Payload selection	Limited cytotoxic screening	AI toxicity–efficacy prediction	QSAR, deep toxicity networks	QSAR databases, PubChem BioAssay	Optimal therapeutic window	Safer payloads for gynecological cancers	[51,52]
Internalization prediction	Cell-line screening	AI modeling of receptor trafficking	Systems biology ML	Cell imaging datasets, systems biology modeling platforms	Improved cellular uptake	Enhanced drug delivery efficiency	[53,54]
Resistance prediction	Post-treatment observation	AI-based resistance pathway modeling	Pathway AI, digital twins	Longitudinal transcriptomic datasets, digital twin frameworks	Early resistance detection	Personalized conjugate redesign	[13,55]
Tumor heterogeneity handling	Single-target design	AI-driven multi-target optimization	Ensemble learning models	Single-cell RNA-seq datasets, TCGA	Broader tumor coverage	Better response in heterogeneous ovarian tumors	[2,21,22]
Personalized conjugate design	Population-based therapy	Patient-specific AI modeling	Precision oncology AI	Precision oncology datasets, multi-omics repositories	Individualized conjugates	Personalized gynecological cancer therapy	[4,56]
Clinical translation support	Long trial cycles	AI-assisted trial stratification	Predictive analytics	Clinical trial registries, predictive analytics platforms	Faster clinical success	Reduced trial failure	[57,58]

**Table 4:** Comparative overview of therapeutic strategies in gynecological cancer treatment.

Parameter	Conventional Chemotherapy	Traditional Targeted Conjugates	AI-Guided High-Affinity Biomolecule–Drug Conjugates	References
Target specificity	Non-selective cytotoxicity	Antigen-dependent but variable	Potential for affinity-optimized and context-specific targeting	[5,6,18]
Design strategy	Empirical, trial-based	Semi-rational molecular design	Data-driven and predictive AI-assisted modeling	[4,13,49]
Off-target toxicity	High systemic toxicity	Reduced but still present	Potential reduction through optimized binding and controlled release	[12,51]
Tumor heterogeneity handling	Limited capability	Restricted by single-target dependence	AI-assisted multi-parameter target adaptation and stratification	[2,21,22]
Resistance prediction	Primarily post-treatment observation	Limited predictive capability	Emerging AI-based prediction of resistance-associated pathways	[13,55]
Internalization efficiency	Uncontrolled	Variable and target-dependent	Computational optimization of uptake and intracellular trafficking	[53,54]
Payload release control	Non-specific systemic exposure	Linker-dependent release	AI-assisted prediction of spatiotemporal release behavior	[12,50]
Personalization potential	Minimal	Limited	Potential for patient-specific conjugate optimization	[4,56]
Clinical translation efficiency	Variable and often limited by toxicity	Moderate translational success	Emerging translational potential with limited prospective clinical validation	[57,58]
Development workflow	Sequential experimental screening	Iterative molecular optimization	AI-assisted virtual screening and multivariable optimization	[20,36]
Validation status	Clinically established	Clinically established in selected settings	Predominantly preclinical and early translational stage	[13,57,58]
Future scalability	Limited adaptability	Moderate scalability	Potentially scalable through continuous data-driven learning	[20,36]

## Data Availability

No new data was created or analyzed in this study. Data sharing is not applicable to this article.

## Abbreviations

ADC	Antibody–Drug Conjugate
ADMET	Absorption, Distribution, Metabolism, Excretion, and Toxicity
AI	Artificial Intelligence
API	Active Pharmaceutical Ingredient
BDCs	Biomolecule–Drug Conjugates
CAR	Chimeric Antigen Receptor
CNN	Convolutional Neural Network
3D-CRT	Three-Dimensional Conformal Radiotherapy
DL	Deep Learning
DNA	Deoxyribonucleic Acid
GNN	Graph Neural Network
ML	Machine Learning
MoA	Mechanism of Action
MUC16	Mucin 16
Omics	Genomics, Proteomics, Transcriptomics, etc.
OS	Overall Survival
PD	Pharmacodynamics
PDCs	Peptide–Drug Conjugates
PFS	Progression-Free Survival
PK	Pharmacokinetics
RNA	Ribonucleic Acid
RNN	Recurrent Neural Network
scFv	Single-chain Fragment Variable
SELEX	Systematic Evolution of Ligands by Exponential Enrichment
TME	Tumor Microenvironment
VEGF	Vascular Endothelial Growth Factor
VMAT	Volumetric Modulated Arc Therapy.
